# Probabilistic Aseismic Performance Assessment of Rubber–Sand–Concrete Tunnel Linings Considering Spatial Variability of Rock Mass

**DOI:** 10.3390/ma19091741

**Published:** 2026-04-24

**Authors:** Kaichen Li, Xiancheng Mei, Baiyi Li, Hao Sheng, Zhen Cui, Yiheng Wang, Hegao Wu, Tao Wang

**Affiliations:** 1 State Key Laboratory of Water Resources Engineering and Management, Wuhan University, Wuhan 430072, China; likaichen@hrbeu.edu.cn (K.L.); hgwu@whu.edu.cn (H.W.); htwang@whu.edu.cn (T.W.); 2 State Key Laboratory of Intelligent Construction and Healthy Operation and Maintenance of Deep Underground Engineering, Xuzhou 221116, China; libaiyi@cumt.edu.cn; 3 State Key Laboratory of Geomechanics and Geotechnical Engineering Safety, Institute of Rock and Soil Mechanics, Chinese Academy of Sciences, Wuhan 430071, China; zcui@whrsm.ac.cn (Z.C.); wangyiheng24@mails.ucas.ac.cn (Y.W.); 4 Yantai Research Institute, Harbin Engineering University, Yantai 264006, China; 5 Hubei Key Laboratory of Roadway Bridge and Structure Engineering, School of Civil Engineering and Architecture, Wuhan University of Technology, Wuhan 430070, China; hsheng@whut.edu.cn

**Keywords:** tunnel engineering, rubber–sand–concrete, aseismic performance, spatial variability, random field

## Abstract

In tunnel engineering, the integration of aseismic materials and structural designs has become a prevalent strategy to reduce earthquake-induced damage. However, previous studies on the seismic performance of tunnel structures predominantly employed deterministic methods, overlooking the spatial variability of the surrounding rock mass. This oversight often leads to an overestimation of structural performance, posing potential risks to the project. This study develops a probabilistic framework based on random field theory to evaluate the aseismic performance of tunnel linings incorporating a rubber–sand–concrete (RSC) constrained damping layer. The analysis systematically evaluates the aseismic performance of RSC across varying peak ground acceleration (PGA) levels and tunnel depth conditions. The findings are compared with results from traditional deterministic approaches. The probabilistic analysis indicates the following: (1) a reduction of approximately 70% in the dispersion of maximum principal stresses across various PGAs; (2) a decrease in RSC’s aseismic performance with greater burial depths, though it remains substantial overall, and (3) a reduction in the failure probability from 31.8% to 16.3% at PGA = 1.2 g. Furthermore, deterministic methods tend to produce overly optimistic estimates of tunnel aseismic performance, highlighting the need for probabilistic analysis.

## 1. Introduction

Tunnels are frequently severely damaged or compromised by earthquakes [1,2,3,4,5,6]. Earthquake-induced tunnel damage has been widely documented, resulting in significant economic losses, property damage, and threats to public safety [7,8,9,10,11]. As a result, establishing effective seismic countermeasures and comprehensive performance evaluation methods has become imperative for ensuring tunnel safety [12,13,14]. Recent advancements in flexible damping technologies, which offer energy dissipation capabilities, have gained prominence in mitigating earthquake-induced damage in tunnel engineering [15,16,17,18]. However, a significant research gap persists in the accurate performance evaluation of these damping technologies. The current state of practice predominantly relies on deterministic analytical methods that utilize mean property values, thereby failing to adequately incorporate the inherent uncertainties in geological conditions and mechanical parameters. This methodological limitation results in incomplete assessments of aseismic performance. A probabilistic framework that systematically accounts for geological and mechanical uncertainties is therefore essential for reliable performance evaluation of flexible damping technologies in seismic applications. Such an approach would provide a more realistic representation of actual field conditions and enable more informed decision-making in tunnel aseismic design and retrofitting projects.

Recent advances in tunnel aseismic protection have focused on flexible damping technologies [19,20,21,22,23], particularly rubber–sand–concrete (RSC) composites. RSC-based damping systems offer a unique combination of engineering and environmental advantages: they provide exceptional energy dissipation through constrained layer damping mechanisms, while also exhibiting favorable dynamic properties such as improved deformability, reduced stiffness, and enhanced vibration attenuation under dynamic loading. At the same time, they enable the sustainable reuse of waste tire rubber [24,25]. This dual functionality represents an improvement over conventional isolation materials, as RSC composites can maintain both effective damping performance and reliable structural support across different seismic intensity levels and geological conditions.

In recent years, engineering risk analysis has gained increasing attention [26,27,28,29,30,31,32,33,34]. However, previous aseismic assessments often assumed deterministic rock behavior, neglecting the critical influence of geotechnical variability on engineering safety. Accounting for this variability improves risk evaluation and enhances the reliability and cost-effectiveness of aseismic design. Geotechnical uncertainty arises from three main sources: inherent spatial variability, measurement errors, and transformation uncertainty. While inherent variability is intrinsic, measurement and transformation errors can be reduced through improved equipment and training [35]. The spatial variability of soil can be decomposed into a smoothly varying trend function *t*(*z*) and a fluctuating component *w*(*z*), as follows:

(1) $$\xi \left(z\right)=t\left(z\right)+w\left(z\right)$$

Currently, the spatial variability of research parameters is commonly characterized explicitly as a random field, based on random field theory [36]. Several methods have been developed to generate random fields, including Cholesky decomposition [37,38,39], Karhunen–Loève (K-L) expansion [40], and spectral representation [41,42]. In slope stability analysis, researchers [43,44,45,46] have demonstrated its significant influence on failure mechanisms. Similarly, studies on foundation bearing capacity [28,47] and settlement analysis [48,49,50,51] have highlighted the critical role of spatial variability. Internationally, the consideration of inherent variability in geotechnical engineering has gained attention from several organizations. For example, ISO 2394 [52] introduced Annex D, “Reliability of Geotechnical Structures,” and similar updates were adopted by the Canadian Highway Bridge Design Code [53]. The performance of underground tunnels is significantly influenced by the properties of the surrounding soil and rock. Many studies have examined the impact of spatial variability in soil properties on tunnel performance [54,55,56,57]. Therefore, accounting for the inherent variability of the rock mass is essential when evaluating the aseismic performance of isolation layers such as RSC.

This study addresses a gap in tunnel aseismic design by developing a probabilistic framework for evaluating the aseismic performance of tunnels with RSC damping layers while explicitly incorporating the spatial variability of rock mass. Although probabilistic approaches based on random field theory and Monte Carlo simulation have been widely applied in geotechnical engineering, their application to the aseismic performance assessment of RSC-protected tunnel structures remains limited. In this study, the Monte Carlo method (MCM) is used to analyze multiple aseismic performance indicators, including maximum principal stress distribution, the probability of plastic strain zones, and seismic failure grades of tunnels along the Sichuan–Tibet Railway under different seismic intensities (PGA 0.1–1.2 g) and burial depths. By comparing the results with those obtained from conventional deterministic analysis, the study not only demonstrates the energy-dissipation effectiveness of RSC, but also quantifies the influence of rock mass spatial variability on tunnel aseismic resilience. The proposed framework therefore provides a probabilistic basis for the design and assessment of constrained damping structures in spatially variable geological environments.

## 2. Methodology

### 2.1. Random Field Theory

#### 2.1.1. Autocorrelation Function

A key component of random field theory is the autocorrelation function (ACF), which defines the relationship between two points in the random field based on their spatial separation. In field testing, direct measurements are often expensive and limited in scope, so theoretical models of the ACF are commonly used to represent rock mass properties [58]. Various forms of theoretical ACF exist, with the exponential ACF being the most widely used, which is expressed as follows:

(2) $$\rho \left(\tau_{x},\tau_{y}\right)=\exp\left[-2\sqrt{{\left(\frac{\tau_{x}}{\delta_{h}}\right)}^{2}+{\left(\frac{\tau_{y}}{\delta_{v}}\right)}^{2}}\right]$$

where $\tau_{x}$ and $\tau_{y}$ are the horizontal and vertical distances between any two points in the random field; $\delta_{h}$ and $\delta_{v}$ are the horizontal and vertical scales of fluctuation (SOFs), respectively. Generally, rock mass properties exhibit stronger correlations for points that are closer together and weaker correlations as the distance increases. This phenomenon is consistent with common observations in natural rock formations, where properties show a strong correlation over short distances and a weaker correlation over larger distances.

#### 2.1.2. Scales of Fluctuation

In practice, SOFs are often unknown, and their estimation can be challenging. Estimating the values of $\delta_{h}$ and $\delta_{v}$ typically necessitates multiple closely spaced data points, for example, multiple data points gathered from a single site. To address this issue, the maximum likelihood estimation (MLE) method is commonly applied [26].

This method allows for the estimation of $\delta_{h}$ and $\delta_{v}$ based on the uniaxial compressive strength ($\sigma_{ci}$) obtained from a limited number of site-collected data points. The calculated $\delta_{h}$ and $\delta_{v}$ can be applied not only to simulate random fields for $\sigma_{ci}$, but also for simulating random fields for other rock parameters. Fenton et al. [59] argued that the spatial variability of various soil and rock parameters is largely consistent, as these parameters are primarily influenced by weathering patterns, stresses, and geological formation histories. Similarly, this study posits that the SOFs of different rock parameters are generally consistent.

$\sigma_{ci}$ can be denoted by $\left\{\sigma_{ci}\left(x_{i},y_{i},z_{i}\right):i=1,\ldots ,m\right\}$, where $\left(x_{i},y_{i},z_{i}\right)$ is the coordinate of location for the *i*-th rock core, and *m* is the total number of data points. To avoid negative parameter values during sampling, a lognormal distribution is typically assumed for all parameters. The mean value and standard deviation of $\ln\left(\sigma_{ci}\left(x,y,z\right)\right)$ can be estimated as:

(3) $$\mu_{\ln\left(\sigma_{ci}\right)}\approx \frac{1}{m}\sum_{i=1}^{m}\ln\left[\sigma_{ci}\left(x_{i},y_{i},z_{i}\right)\right]$$

(4) $$\sigma_{\ln\left(\sigma_{ci}\right)}\approx \sqrt{\frac{1}{m}\sum_{i=1}^{m}\ln{\left\{\left[\sigma_{ci}\left(x_{i},y_{i},z_{i}\right)\right]-\mu_{\ln\left(\sigma_{ci}\right)}\right\}}^{2}}$$

Assuming the SOF of the two horizontal directions (*x*, *y*) are identical to $\delta_{h}$, the covariance matrix $\left\{\ln\left[\sigma_{ci}\left(x_{i},y_{i},z_{i}\right)\right]:i=1,\ldots ,m\right\}$ is as follows:

(5) $$\sum \left(\delta_{h},\delta_{v}\right)=\sigma_{\ln\left(\sigma_{ci}\right)}^{2}\left[\begin{array}{cccc} 1 & \exp\left(-2\sqrt{{\left(\frac{\Delta h_{1,2}}{\delta_{h}}\right)}^{2}+{\left(\frac{\Delta z_{1,2}}{\delta_{v}}\right)}^{2}}\right) & \ldots & \exp\left(-2\sqrt{{\left(\frac{\Delta h_{1,m}}{\delta_{h}}\right)}^{2}+{\left(\frac{\Delta z_{1,m}}{\delta_{v}}\right)}^{2}}\right)\\ \exp\left(-2\sqrt{{\left(\frac{\Delta h_{2,1}}{\delta_{h}}\right)}^{2}+{\left(\frac{\Delta z_{2,1}}{\delta_{v}}\right)}^{2}}\right) & 1 & \ldots & \exp\left(-2\sqrt{{\left(\frac{\Delta h_{2,m}}{\delta_{h}}\right)}^{2}+{\left(\frac{\Delta z_{2,m}}{\delta_{v}}\right)}^{2}}\right)\\ \vdots & \vdots & \ddots & \vdots \\ \exp\left(-2\sqrt{{\left(\frac{\Delta h_{m,1}}{\delta_{h}}\right)}^{2}+{\left(\frac{\Delta z_{m,1}}{\delta_{v}}\right)}^{2}}\right) & \exp\left(-2\sqrt{{\left(\frac{\Delta h_{m,2}}{\delta_{h}}\right)}^{2}+{\left(\frac{\Delta z_{m,2}}{\delta_{v}}\right)}^{2}}\right) & \ldots & 1 \end{array}\right]$$

where $\Delta h_{i,j}=\sqrt{{\left(x_{i}-x_{j}\right)}^{2}+{\left(y_{i}-y_{j}\right)}^{2}}$, $\Delta z_{i,j}=\left|z_{i}-z_{j}\right|$ are the horizontal and vertical distances, respectively, between the *i*-th and *j*-th rock cores. The likelihood function $L\left\{\delta_{h},\delta_{v}\left|\ln\left[\sigma_{ci}\left(x_{i},y_{i},z_{i}\right)\right]:i=1,\ldots ,m\right.\right\}$ of $\delta_{h}$ and $\delta_{v}$ is:

(6) $$\begin{array}{l} L\left\{\delta_{h},\delta_{v}\left|\ln\left[\sigma_{ci}\left(x_{i},y_{i},z_{i}\right)\right]:i=1,\ldots ,m\right.\right\}\\ =\frac{1}{{\left(2\pi \right)}^{m/2}\sqrt{\det\left(\sum \left(\delta_{h},\delta_{v}\right)\right)}}\exp\left\{-\frac{1}{2}{\left[\begin{array}{c} \ln\left[\sigma_{ci}\left(x_{1},y_{1},z_{1}\right)\right]-\mu_{\ln\left(\sigma_{ci}\right)}\\ \ln\left[\sigma_{ci}\left(x_{2},y_{2},z_{2}\right)\right]-\mu_{\ln\left(\sigma_{ci}\right)}\\ \vdots \\ \ln\left[\sigma_{ci}\left(x_{m},y_{m},z_{m}\right)\right]-\mu_{\ln\left(\sigma_{ci}\right)} \end{array}\right]}^{T}\times {\sum \left(\delta_{h},\delta_{v}\right)}^{-1}\left[\begin{array}{c} \ln\left[\sigma_{ci}\left(x_{1},y_{1},z_{1}\right)\right]-\mu_{\ln\left(\sigma_{ci}\right)}\\ \ln\left[\sigma_{ci}\left(x_{2},y_{2},z_{2}\right)\right]-\mu_{\ln\left(\sigma_{ci}\right)}\\ \vdots \\ \ln\left[\sigma_{ci}\left(x_{m},y_{m},z_{m}\right)\right]-\mu_{\ln\left(\sigma_{ci}\right)} \end{array}\right]\right\} \end{array}$$

The maximum value of $L\left\{\delta_{h},\delta_{v}\left|\ln\left[\sigma_{ci}\left(x_{i},y_{i},z_{i}\right)\right]:i=1,\ldots ,m\right.\right\}$ represents the maximum likelihood estimate, which serves as the optimal solution for $\delta_{h}$ and $\delta_{v}$.

#### 2.1.3. Construction of Random Field

The theoretical ACF of the project can be derived by substituting the results of $\delta_{h}$ and $\delta_{v}$ into Equation (2). Based on this, the lognormal random field *G* can be obtained by the K-L expansion method:

(7) $$G=\sum_{t=1}^{M_{k}}\sqrt{\lambda_{t}}\xi_{t}\varphi_{t}\left(x\right)$$

where $\xi_{t}$ is a set of independent standard normal distribution random variables; $\lambda_{t}$ and $\varphi_{t}\left(x\right)$ are the eigenvalues and eigenfunctions of the ACF, respectively; and *M_k_* is the number of terms of the series expansion.

In summary, the random field of rock parameter *X* (in this study, *X* includes *E*, *ν*, *c,* and *φ*) can be generated by the mean value $\mu_{X}$, coefficient of variation COV*_X_*, and SOF*_X_* ($\delta_{hX}$, $\delta_{vX}$). The mean value and standard deviation of the logarithm of *X*’s random field are calculated by:

(8) $$\mu_{\ln X}=\ln\left(\mu_{X}/\sqrt{1+{\mathrm{COV}}_{X}^{2}}\right)$$

(9) $$\sigma_{\ln X}=\sqrt{\ln\left(1+{\mathrm{COV}}_{X}^{2}\right)}$$

The value of the random field $F_{X}\left(x_{i},y_{i}\right)$ at any position $\left(x_{i},y_{i}\right)$ in space is:

(10) $$F_{X}\left(x_{i},y_{i}\right)=\exp\left[\mu_{\ln X}+\sigma_{\ln X}\cdot G\left(x_{i},y_{i}\right)\right]$$

Shear strength parameters *c* and *φ* are typically negatively correlated. Therefore, the correlation between *c* and *φ* is expressed using the cross-correlation coefficient $\rho_{c,\varphi }$. The cross-correlation coefficient ${\rho^{\prime }}_{c,\varphi }$ between ln*c* and ln*φ* can be obtained as follows:

(11) $${\rho^{\prime }}_{c,\varphi }=\frac{\ln\left(1+\rho_{c,\varphi }{\mathrm{COV}}_{c}{\mathrm{COV}}_{\varphi }\right)}{\sqrt{\ln\left(1+{\mathrm{COV}}_{c}^{2}\right)\ln\left(1+{\mathrm{COV}}_{\varphi }^{2}\right)}}$$

The cross-correlated lognormally distributed random of *c* and *φ* can be obtained with the following equations:

(12) $$F_{c}\left(x_{i},y_{i}\right)=\exp\left[\mu_{\ln c}+\sigma_{\ln c}\cdot G_{c}\left(x_{i},y_{i}\right)\right]$$

(13) $$F_{\varphi }\left(x_{i},y_{i}\right)=\exp\left[\mu_{\ln\varphi }+\sigma_{\ln\varphi }\left(G_{c}\left(x_{i},y_{i}\right){\rho^{\prime }}_{c,\varphi }+G_{\varphi }\left(x_{i},y_{i}\right)\sqrt{1-\rho_{c,\varphi }^{\prime 2}}\right)\right]$$

### 2.2. Implementation Procedure

**Figure 1:**
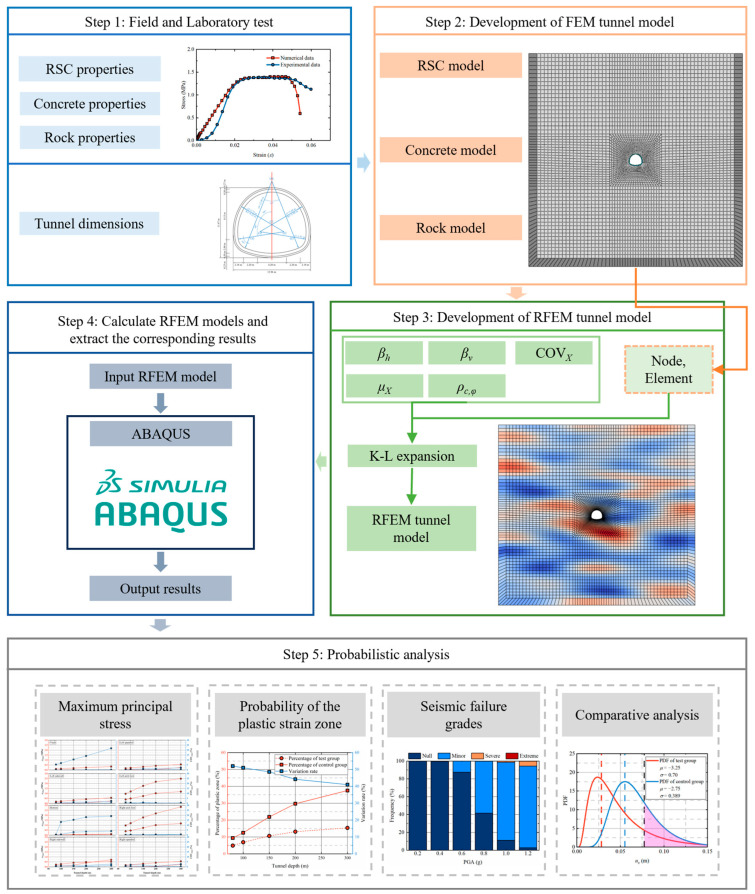
Workflow for the implementation of the probabilistic analysis.

This subsection presents the overall numerical and analytical framework adopted in this study for evaluating the aseismic performance of tunnels with RSC damping layers. ABAQUS 6.14 version was employed as the finite element platform because it can effectively simulate the nonlinear dynamic response of tunnel structures under seismic loading, while allowing detailed representation of material properties, structural configuration, and boundary conditions. Figure 1 illustrates the schematic workflow of this study, which consists of five key steps, which are summarized as follows:

Step 1: Determine statistical parameters, including tunnel dimensions, concrete and RSC properties, and the rock properties, based on engineering data and laboratory tests.

Step 2: A tunnel model was developed using the finite element method (FEM) and ABAQUS software.

Step 3: Generate *N_sim_* sets of random fields using the K-L method described in Section 2.1 to perform the MCM and replace the Mohr–Coulomb parameter values in the original tunnel model with those derived from the random fields.

Step 4: Calculate the *N_sim_* sets of RFEMs by ABAQUS software and extract the corresponding results.

Step 5: Perform a probabilistic analysis of the results for assessment of the tunnel’s aseismic performance.

## 3. Development of Probabilistic Analysis

The aseismic improvement capabilities of RSC in tunnel applications, as demonstrated in previous analyses, necessitate a probabilistic assessment to inform the engineering design of constrained damping configurations. In the initial phase of the engineering investigation, the $\mu_{X}$, COV*_X_*, SOF*_X_* ($\delta_{hX}$, $\delta_{vX}$), and $\rho_{c,\varphi }$ of the rock mass’s *E*, *ν*, *c*, and *φ* were computed, followed by the establishment of the random field model for the project. Subsequently, the random field model for the project was established. This model generated multiple samples for computational analysis of RSC’s impact on tunnel structural behavior, focusing on the distribution of maximum principal stress and the probability of plastic strain zones under varying PGAs and tunnel depths. For quantitative seismic impact assessment, the relative deformation rate *η* was introduced, which was defined as:

(14) $$\eta =\frac{\sqrt{{\left|H_{1}-H_{2}\right|}^{2}+{\left|V_{1}-V_{2}\right|}^{2}}}{l}$$

where *H*_1_ and *H*_2_ are the horizontal displacements of the right arch feet and left spandrel, respectively, while *V*_1_ and *V*_2_ denote the vertical displacements of the aforementioned points. The parameter *l* is the distance between the right arch feet and the left spandrel. According to the Chinese Code [60], tunnel structural performance is categorized into different seismic failure grades based on *η*, as outlined in Table 1. By analyzing the frequency of occurrence of each failure grade, the aseismic performance of RSC under varying conditions can be evaluated.

### 3.1. Field Information

**Figure 2:**
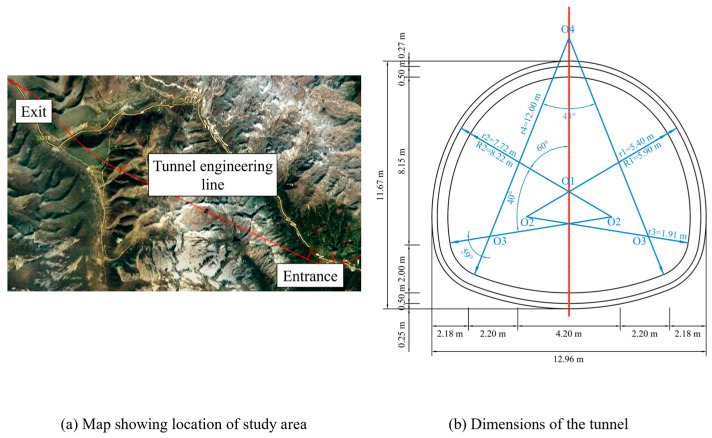
Field information.

This research was conducted on a railway tunnel located in a seismically active mountainous region. The tunnel traverses a high-altitude mountain range and enters a plateau area, featuring significant portal elevation differences (Figure 2a). It has a maximum overburden depth of 1235 m and adopts a four-centered circular cross-section lined with C30 concrete (Figure 2b).

**Figure 3:**
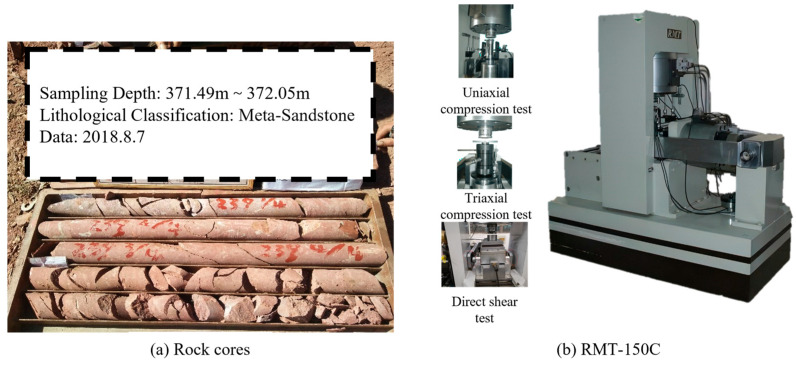
Rock cores and RMT-150C.

To determine the $\mu_{X}$, COV*_X_*, SOF*_X_* ($\delta_{hX}$, $\delta_{vX}$), and $\rho_{c,\varphi }$ of rock mass’s *E*, *ν*, *c*, and *φ*, as well as other mechanical properties of the surrounding rock and lining structure, extensive field and laboratory testing was conducted. A total of 54 boreholes, totaling 4461.74 m, were drilled at the site. As shown in Figure 3a, core samples were systematically documented post-drilling, with key parameters—including borehole identification number, sampling depth, and lithological classification—recorded and photographically archived for subsequent analysis. A total of 185 cores were obtained and used for various laboratory mechanical experiments, utilizing the RMT-150C servo-controlled testing machine (Figure 3b). These included 428 uniaxial compression tests, 210 direct shear tests, and 210 triaxial compression tests.

Through laboratory and field testing across multiple loading conditions, reliable correlations were established between experimental measurements and numerical parameters. This rigorous calibration approach, incorporating statistical analysis of test data variability, ensures that the numerical simulations accurately reflect the real-world aseismic performance of tunnel structures.

### 3.2. Calculation Model

**Figure 4:**
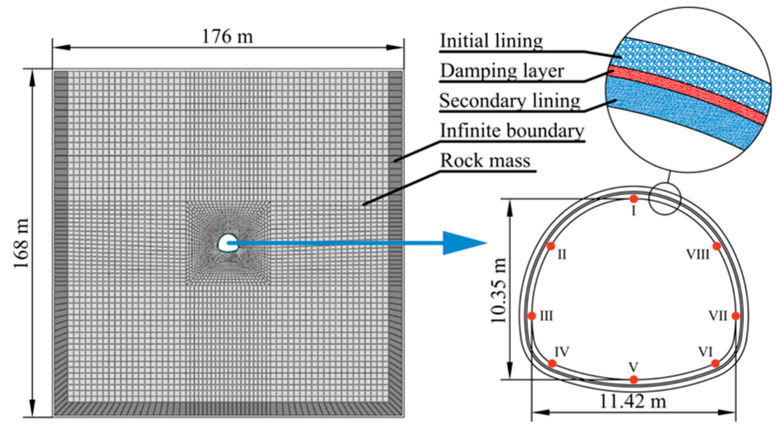
Numerical model.

This study employed a probabilistic approach to evaluate the aseismic performance of RSC using a two-dimensional (2D) finite element model (Figure 4). The numerical framework incorporated the spatial variability of rock mass properties through 500 MCMs, with each realization generating a unique random field distribution. The computational domain measuring 176 m × 168 m was discretized using quadrilateral elements, with infinite boundary elements incorporated to eliminate wave reflection artifacts and ensure accurate dynamic response simulation. The numerical model comprises 7300 quadrilateral elements (8241 nodes). In the present model, the initial vertical stress was determined from the self-weight of the overlying rock mass, i.e., from the product of rock density and burial depth. A separate horizontal in situ stress was not explicitly considered. This simplified stress treatment was adopted to focus on the influence of tunnel depth while maintaining computational efficiency in the probabilistic analysis, although a more complete in situ stress representation may be considered in future work. The rock-lining interface was simulated through a combined Lagrange multiplier–penalty approach, assigning the concrete lining as the master surface and the surrounding rock as the slave surface. The normal interface behavior follows Hooke’s law, while the tangential response is characterized by Coulomb friction with a coefficient of 0.5. The tunnel geometry features a four-centered circular cross-section (11.42 m span × 10.15 m height) at a depth of 80 m, modelled with a composite lining system comprising: (1) 0.35 m thick primary and secondary concrete layers and (2) a 0.175 m RSC damping layer. Response monitoring was implemented at the tunnel’s inner surface of eight critical locations (I–VIII) across the vault, spandrels, sidewalls, arch feet, and invert to capture the full structural behavior under seismic excitation.

A dynamic implicit time history analysis methodology was adopted to assess the aseismic resistance of RSC. The computational sequence began by establishing stress equilibrium states under gravitational loads for tunnels at varying burial depths. These geostatic stress distributions were then applied as initial boundary conditions in the dynamic analysis.

### 3.3. Material Parameters

**Figure 5:**
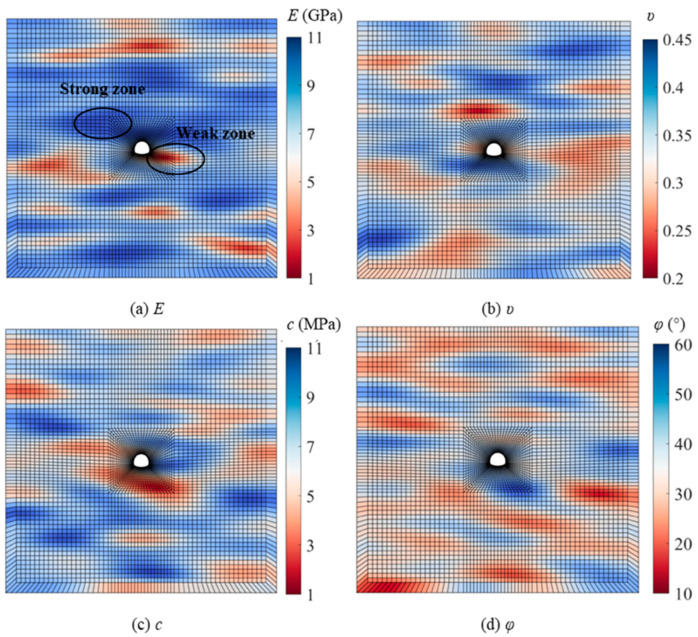
Typical random field of rock mass.

In this study, 428 data points for uniaxial compressive strength were obtained from rock cores collected from 51 boreholes. The method described in Section 2.1.2 was used to calculate the SOF*_X_* ($\delta_{hX}$, $\delta_{vX}$) of the rock mass based on the uniaxial compressive strength data and the spatial coordinates of the core samples. Additionally, $\mu_{X}$, COV*_X_*, and $\rho_{c,\varphi }$ of the rock mass were further derived from the results of laboratory experiments. The rock mass density was determined as 2300 kg/m^3^, with additional stochastic parameters detailed in Table 2. A typical realization of parameter distributions (*E*, *ν*, *c*, *φ*) is visualized in Figure 5, demonstrating the characteristic spatial variability and the negative correlation between *c* and *φ*.

The material parameters for both C30 concrete and the RSC composite are presented in Table 3. For the C30 lining concrete, the well-established Concrete Damaged Plasticity (CDP) model, developed by Lee and Fenves [61], was adopted to capture the nonlinear behavior of concrete. The uniaxial tensile and compressive damage evolution curves for C30 were derived in accordance with the Chinese Code [62]. This approach ensures that the numerical model accurately reflects the mechanical degradation processes in tunnel lining concrete under seismic loading conditions.

The failure characteristics of RSC were quantified using the energy-based damage evolution theory developed by Hillerborg et al. [63], which describes progressive material degradation through fracture energy dissipation. This model was adopted because it provides a practical macroscopic representation of the mechanical response of RSC within the present finite element framework and is compatible with the adopted numerical formulation. The critical fracture energy is expressed as:

(15) $$G_{f}=\int_{{\bar{\varepsilon }}_{0}^{\mathrm{pl}}}^{{\bar{\varepsilon }}_{f}^{\mathrm{pl}}}\sigma_{y}d{\bar{\varepsilon }}^{\mathrm{pl}}=\int_{0}^{{\bar{u}}_{f}^{\mathrm{pl}}}\sigma_{y}d{\bar{u}}^{\mathrm{pl}}$$

where $\sigma_{y}$ represents stress, while ${\bar{\varepsilon }}^{\mathrm{pl}}$ denotes equivalent plastic strain. ${\bar{\varepsilon }}_{0}^{\mathrm{pl}}$ and ${\bar{\varepsilon }}_{f}^{\mathrm{pl}}$ correspond to the equivalent plastic strain at damage initiation and complete failure, respectively. ${\bar{u}}_{f}^{\mathrm{pl}}$ is the equivalent plastic displacement.

**Figure 6:**
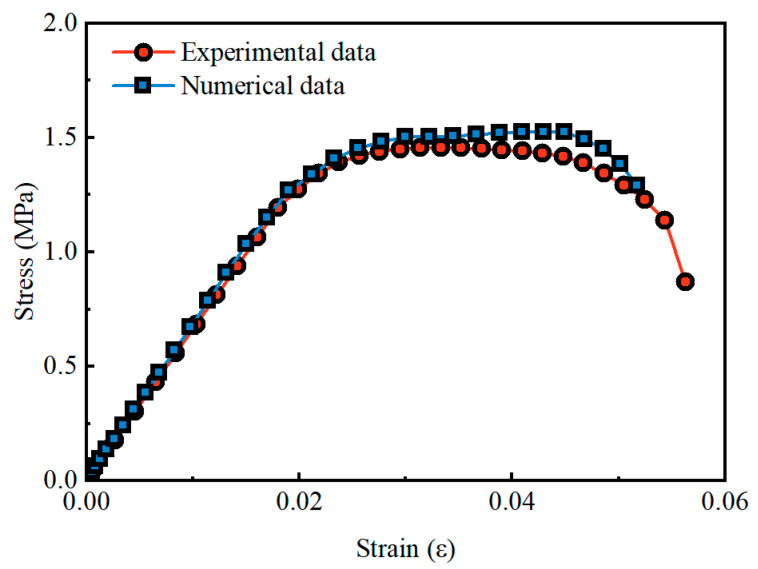
Numerical and experimental results for RSC.

The material parameters for RSC in aseismic performance analysis were validated through numerical simulations replicating the uniaxial compression test that was conducted by Mei et al. [24]. As shown in Figure 6, the numerical models demonstrate close agreement with experimental measurements in stress–strain response, confirming that the calibrated parameters effectively capture the essential mechanical characteristics of RSC.

### 3.4. Calculation Program

**Figure 7:**
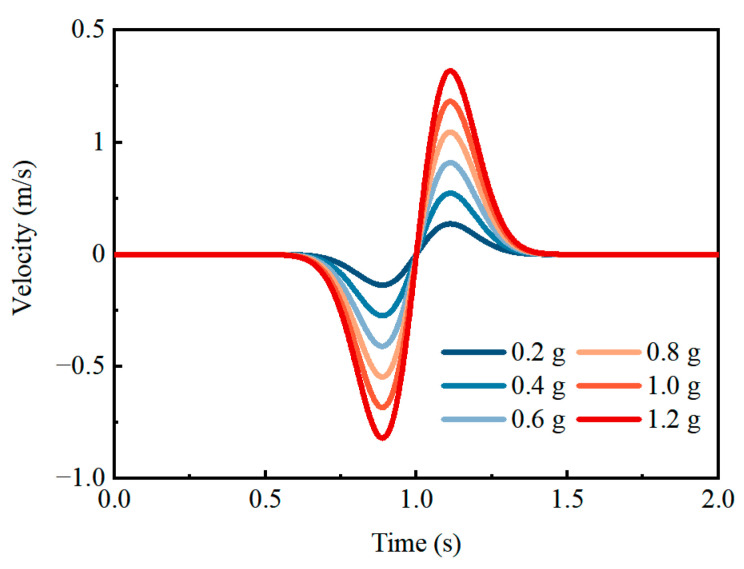
Ricker’s wavelets with different PGAs.

Table 4 presents 22 sets of random field analyses for varying PGAs and tunnel depths. These sets systematically evaluate RSC performance across (1) six seismic intensity levels (PGA = 0.2–1.2 g), corresponding to a 1% probability of exceedance over 100 years of seismic events, and (2) five characteristic tunnel depths (80 m, 100 m, 150 m, 200 m, and 300 m). Computational efficiency was enhanced through the use of Ricker wavelet excitation (Figure 7). This probabilistic analysis design enables a characterization of RSC’s PGA- and depth-dependent seismic mitigation effectiveness, while accounting for the spatial variability of rock mass.

## 4. Results

### 4.1. Aseismic Performance Under Different PGAs

Stress concentration plays an important role in the aseismic performance of tunnel structures, as local stress amplification may lead to damage initiation in critical regions. Therefore, this subsection first examines the stress response of the tunnel lining, and the following figures are used to illustrate the influence of the RSC damping layer on stress concentration.

**Figure 8:**
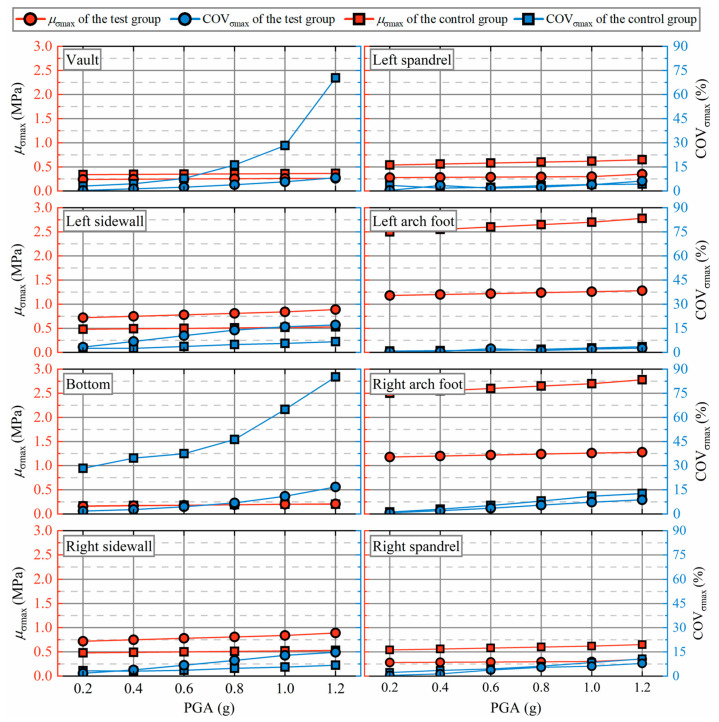
Effect of PGA on the maximum principal stress.

As illustrated in Figure 8, the mean value (*μ*_σmax_) and coefficient of variation (COV_σmax_) of the maximum principal stress at each monitoring point vary with increasing PGA. The results demonstrate that the RSC damping layer not only reduces *μ*_σmax_ but also substantially narrows its dispersion. As the PGA increases, *μ*_σmax_ at each monitoring point remains relatively stable, with the RSC damping layer effectively reducing *μ*_σmax_ during excitation. The reduction in *μ*_σmax_ is approximately 50% at both the spandrel and arch foot of the lining. While stress concentration tends to occur at the arch foot, the RSC damping layer significantly mitigates this effect. Although COV_σmax_ increases with the PGA at each monitoring point, the rate of increase is lower in the test groups compared to the control groups. Specifically, at the vault in the test group, when the PGA is 1.2 g, COV_σmax_ is only 11.8% of that observed in the control group. Similarly, at the bottom of the test group, COV_σmax_ is only 9.3% of that in the control group. For the remaining monitoring points, the reduction in COV_σmax_ in the test groups is approximately 70% of that in the control group at each PGA value. Furthermore, a clear trend emerges where higher *μ*_σmax_ values correspond to lower COV_σmax_ values. This can be explained by the fact that when *μ*_σmax_ is high, earthquake loading predominantly influences the formation of maximum principal stress, resulting in more concentrated results. As *μ*_σmax_ decreases, other factors, such as rock mass properties, fault conditions, and in situ stress, become more influential, leading to greater dispersion in the results. In summary, the RSC damping layer reduces uncertainty in the maximum principal stress under varying PGAs.

**Figure 9:**
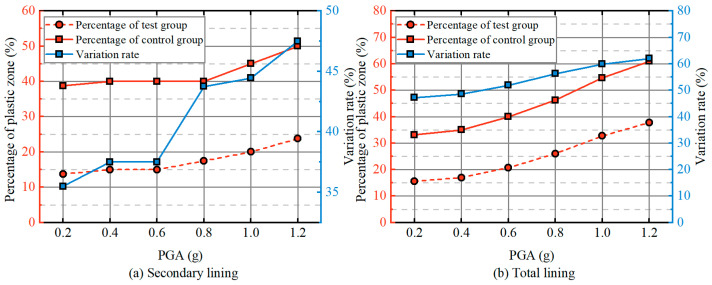
Effect of PGA on the probability of the plastic strain zone.

Figure 9 presents the percentage of the plastic zone in the secondary lining and total lining, along with the corresponding variation rates under different PGAs. For the secondary lining, the percentage of the plastic zone gradually increases. At a PGA of 0.2 g, the plastic zone in the test and control groups accounts for 13.8% and 38.7%, respectively. As the PGA increases to 1.2 g, these values rise to 23.7% and 48.5% for the test and control groups, respectively. The variation rate also increases, from 35.5% to 47.5%. For the total lining, the increase in the percentage of the plastic zone is more pronounced. At a PGA of 0.2 g, plastic damage affects 15.6% and 33.1% of the total lining in the test and control groups, with a variation rate of 47.2%. As the PGA increases, the percentage of the plastic zone grows in both groups, with the variation rate peaking at 61.9% at 1.2 g. These results demonstrate that the RSC damping layer not only improves the ultimate limit state (support capacity) of the tunnel under seismic loading but also enhances its serviceability limit states under the same seismic conditions. Moreover, the improvement in aseismic performance provided by the RSC damping layer becomes more pronounced as the PGA increases.

**Figure 10:**
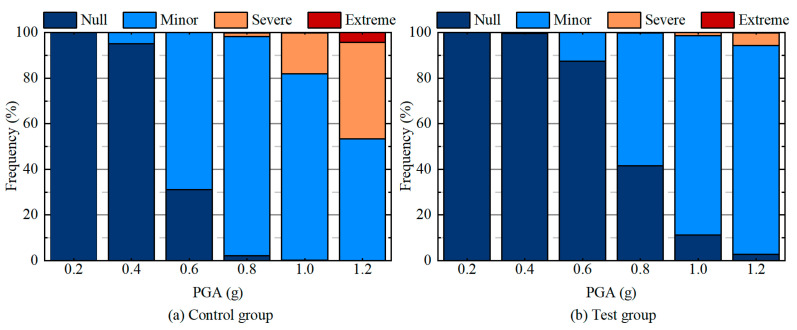
Effect of PGA on the frequency of each seismic failure grade.

*η* for each random field model was evaluated by the criteria listed in Table 1. Figure 10 illustrates the frequency of occurrence for each seismic failure grade. The findings suggest that as the PGA increases, the seismic failure grade for both the test and control groups increases gradually, indicating that the tunnel becomes increasingly vulnerable. When the PGA reaches 0.6 g, the control group begins to experience the “Severe” failure grade. At a PGA of 1.2 g, 42.4% of the control group fails at the “Severe” level, with 4.2% even experiencing an “Extreme” failure grade. This suggests that PGAs between 0.6 g and 1.2 g can cause serious damage to conventional tunnel structures. In contrast, most of the test group remains within the “Null” and “Minor” failure grades under any PGA. Even at a PGA of 1.2 g, only 5.4% of the tunnel structure in the test group reached the “Severe” failure grade. These findings highlight the effectiveness of the RSC damping layer in mitigating seismic damage to tunnel structures, reducing the financial burden of post-earthquake repairs, and enhancing both the safety of individuals and the socio-economic stability of affected regions.

According to the results of the probabilistic analysis, RSC significantly improves tunnel aseismic performance by reducing the dispersion of maximum principal stress by 70% across varying PGAs. Simultaneously, it enhances structural capacity and maintains functionality under extreme loading. The technology’s effectiveness increases proportionally with seismic intensity, offering substantial safety and economic benefits.

### 4.2. Aseismic Performance Under Different Tunnel Depths

**Figure 11:**
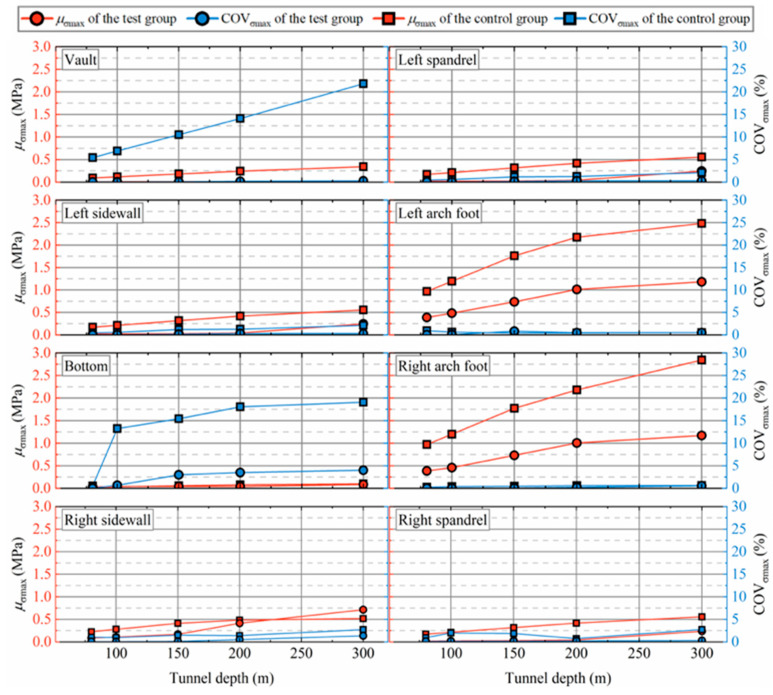
Effect of tunnel depth on the maximum principal stress.

Figure 11 demonstrates the variation of *μ*_σmax_ and COV_σmax_ at each monitoring point with respect to tunnel depth. As the tunnel depth increases, *μ*_σmax_ exhibits notable fluctuations at each monitoring point. The RSC damping layer significantly mitigates *μ*_σmax_ during seismic excitation. Stress concentration is most evident at the arch feet, where *μ*_σmax_ experiences the greatest increase, and the damping layer’s seismic mitigation effect is most pronounced at this location. In the control group, *μ*_σmax_ increases from 1 MPa at a depth of 80 m to 2.5 MPa at 300 m. Conversely, the test group shows a more modest rise in *μ*_σmax_, from 0.387 MPa at 80 m to 1.18 MPa at 300 m, representing only 39.1% and 47.6% of the control group’s values, respectively. For COV_σmax_, variability increases with tunnel depth, with the most significant changes observed at the vault and bottom, where *μ*_σmax_ is lowest. This pattern mirrors the findings in Section 4.1. While COV_σmax_ changes less dramatically at other monitoring points, it remains smaller in the test group compared to the control group. In conclusion, the RSC damping layer reduces both the maximum principal stress and its variability across different tunnel depths. Furthermore, the damping layer’s aseismic performance improves with depth, underscoring its effectiveness in reducing seismic risks in deeper tunnel sections.

**Figure 12:**
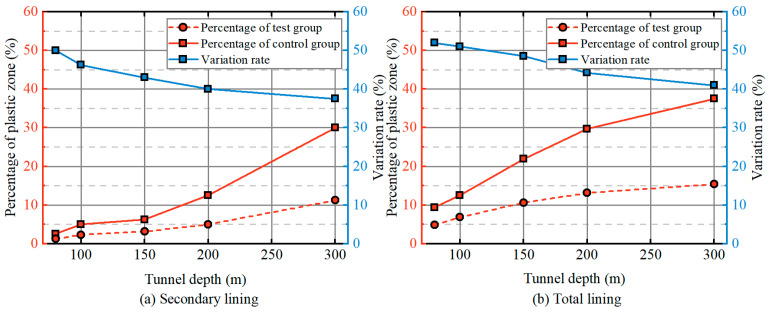
Effect of tunnel depth on the probability of the plastic strain zone.

Figure 12 depicts the percentage of the plastic zone in both the secondary lining and total lining, along with the variation rate, at different tunnel depths. For the secondary lining, the plastic zone percentage increases gradually for depths less than 150 m. However, a significant rise in the plastic zone percentage is observed once the tunnel depth exceeds 150 m. At a depth of 300 m, the control group shows a plastic zone percentage of 30%, while the test group shows only 11.2%. The variation rate decreases from 50% to 37.5%. In the total lining, the plastic zone percentage increases more substantially. At a depth of 80 m, plastic damage affects 4.8% and 9.3% of the total lining in the test and control groups, respectively, with a variation rate of 52%. As the tunnel depth increases, the plastic zone percentage rises in both groups, reaching 15.4% and 37.5% at 300 m, while the variation rate decreases from 52% to 41%. This analysis indicates that the damping layer improves both the ultimate limit state (support capacity) and serviceability limit state (structural functionality) under seismic conditions. However, the degree of improvement diminishes with increasing tunnel depth, although the damping layer’s effectiveness remains notable across all depths.

The probabilistic analysis results confirm that RSC effectively reduces both the magnitude and variability of the maximum principal stress at various tunnel depths, demonstrating its efficacy in deeper sections where seismic risk mitigation is most critical. While the enhancement of the ultimate limit state (structural capacity) and serviceability limit state (functional performance) diminishes with greater depth, the damping layer continues to provide substantial aseismic protection throughout the entire depth range. These findings affirm RSC’s robust performance as a reliable seismic mitigation solution for tunnels under varying conditions, combining stress reduction with consistent protective capacity across all depths.

### 4.3. Comparative Analysis and Discussion

Traditional deterministic tunnel aseismic analysis, which utilizes mean material properties, offers superior computational efficiency (250 s on an i9-14900F system) compared to probabilistic methods (540 s for a single probabilistic analysis and 270,000 s for 500 MCMs). However, it fails to account for the critical geotechnical uncertainties inherent in real-world conditions. The substantial geological accuracy achieved through probabilistic analysis, by characterizing rock variability, justifies its increased computational demands. Enhanced prediction precision provides key advantages for reliably assessing aseismic performance.

**Figure 13:**
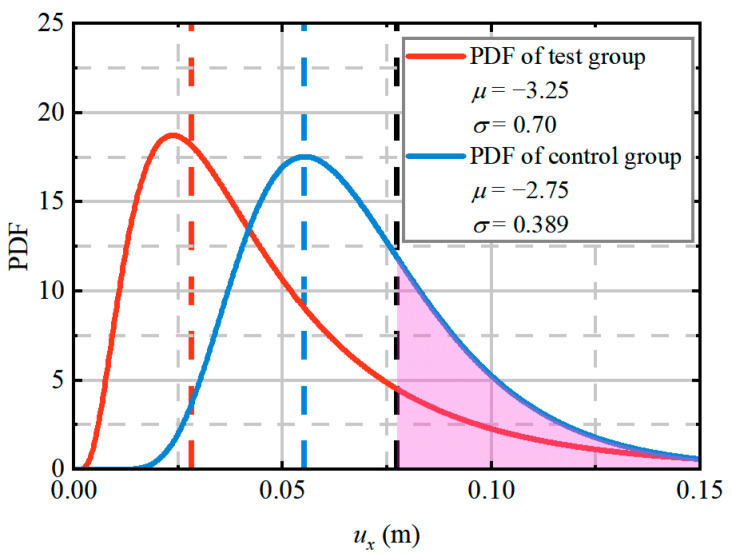
Comparison between deterministic analysis and probabilistic analysis.

A comparison of deterministic and probabilistic analyses for evaluating tunnel wall inward displacement is presented. Following Lü et al. [64], maximum horizontal displacement ($u_{X}$) caused by earthquakes serves as a critical stability indicator, with structural instability occurring when $u_{X}$ surpasses the predefined threshold ($u_{X}^{\max}$), and therefore represents a dynamic-response-based index rather than a static one. This threshold is defined as 1% of the tunnel radius (0.077 m, calculated as 7.73 m × 1%). Figure 13 presents the probability density functions (PDFs) of $u_{X}$ for both the test and control groups under 1.2 g PGA conditions, with both groups exhibiting lognormal distributions. Deterministic analysis produced inward displacements of 0.028 m (test group) and 0.055 m (control group), indicated by dashed lines in Figure 13 (red for the test group, blue for the control group). While probabilistic analysis yielded mean values consistent with these deterministic predictions, the deterministic approach significantly underestimates structural risk by failing to account for outcome variability and extreme values. The $u_{X}$ calculated through deterministic analyses were all found to be below the threshold $u_{X}^{\max}$ (the black dashed line), leading to a consistent classification of the tunnel as safe for that specific operating condition during design. Nevertheless, it is evident from the outcomes of the probabilistic analyses that the $u_{X}$ calculated by both the test and control groups have the potential to exceed $u_{X}^{\max}$, resulting in potential structural failure. This discrepancy is attributed to the assumption of homogenous rock properties in deterministic analysis. In contrast, probabilistic analysis allows for the simulation of weak and strong zones within a single model, as illustrated in Figure 5. It is inevitable that these zones will exhibit different responses to seismic activity, leading to variations in the tunnel lining’s response to earthquakes. Therefore, neglecting uncertainties in input parameters during aseismic analysis can lead to overestimations of tunnel structural response during an earthquake, potentially causing significant failures in field applications.

The failure probability (P*_f_*) was computed as the ratio of failure occurrences to total realizations (*N_sim_* = 500). At a 1.2 g PGA, P*_f_* values of 16.3% (test group) and 31.8% (control group) highlight the substantial safety improvements achieved through the implementation of the RSC damping layer, confirming its superior aseismic performance.

## 5. Discussion

The COV and SOF serve as fundamental parameters governing the spatial variability characterization in random field generation. These parameters exhibit distinct yet complementary influences on rock mass property distributions: COV quantifies the magnitude of parameter dispersion (with higher values indicating greater variability), while SOF describes the spatial correlation length (with smaller values representing more rapid property fluctuations). A comprehensive investigation of COV and SOF effects on RSC aseismic performance is critical for two primary reasons: (1) it enables quantification of uncertainty propagation from geological conditions to structural response; and (2) it facilitates optimization of RSC implementation strategies based on site-specific variability characteristics. Such understanding directly enhances the technology’s engineering applicability by transforming probabilistic analysis results into actionable design guidelines, particularly for critical infrastructure in high-seismicity regions. To systematically evaluate these effects, this study conducts a parametric analysis of RSC performance under extreme loading conditions (300 m overburden depth and PGA = 1.2 g) through comprehensive numerical simulation of $u_{X}$. The COV of *E*, *ν*, *c*, and *φ* are assumed to be 10%, 20%, and 30% when the COV analyses are carried out, and $\delta_{h}$ and $\delta_{v}$ are taken as 50 m and 5 m, respectively. Owing to historical deposition, rock and soil parameters are more correlated in the vertical direction than in the horizontal direction [38]; therefore, it is assumed that $\delta_{h}=10\delta_{v}$ during the SOF analyses. $\delta_{v}$ are assumed to be 5 m, 15 m, and 25 m, respectively, and COV = 30%.

**Figure 14:**
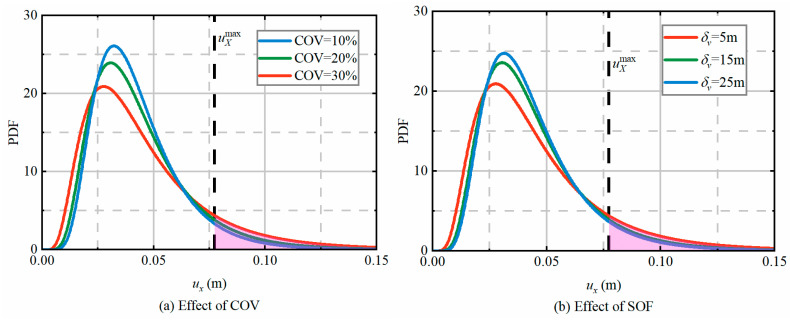
PDF of $u_{X}$ under different COV and SOF.

Figure 14 presents the probability density functions of $u_{X}$ under varying COV and SOF. As shown in Figure 14a, the dispersion of $u_{X}$ progressively increases with higher COV values, accompanied by a rise in failure probability from 5.5% to 11.8%. This demonstrates that greater variability in surrounding rock parameters leads to more pronounced spatial heterogeneity, which substantially impacts tunnel lining behavior and generates increased dispersion in structural response. Conversely, Figure 14b reveals that $u_{X}$’s dispersion decreases with increasing SOF, with the corresponding failure probability declining from 11.4% to 7.7%. This indicates that while SOF influences the spatial variation characteristics of surrounding rock, its effect on aseismic response is less dominant than that of COV.

## 6. Conclusions

This study develops a probabilistic framework for evaluating the aseismic performance of RSC damping layers in tunnel engineering. The main conclusions are as follows:

(1) The probabilistic analysis shows that the RSC damping layer not only reduces the mean maximum principal stress under different PGAs and tunnel depths, but also decreases its variability. In addition, the use of the RSC damping layer improves both the ultimate limit state of support capacity and the serviceability limit states of the tunnel structure. This improvement is mainly attributed to the substantial reduction in the area of the tunnel structure susceptible to plastic damage under all considered conditions. The seismic failure grade of the tunnel is also reduced by the use of the RSC damping layer, indicating its potential to both lower post-earthquake repair costs and enhance structural safety.
(2) Comparative results indicate that deterministic analysis may lead to an overly optimistic assessment of tunnel stability during seismic events because it does not explicitly account for the spatial variability of the surrounding rock mass. In this respect, probabilistic analysis provides a more comprehensive basis for evaluating the aseismic performance of tunnels. Furthermore, the implementation of the RSC damping layer reduces the failure probability of the tunnel structure from 31.8% to 16.8% at a PGA of 1.2 g, which further confirms its favorable aseismic performance.
(3) The aseismic performance of RSC is significantly influenced by the random-field parameters, especially COV and SOF. An increase in COV leads to higher mean response values and greater dispersion, thereby increasing the structural failure risk. By contrast, an increase in SOF tends to reduce the dispersion of the results, although its influence is less pronounced than that of COV.

It should also be noted that the present study mainly focuses on the effects of PGA, tunnel depth, and key random-field parameters on the aseismic performance of tunnels with RSC damping layers. Other factors, such as damping-layer thickness and the stiffness ratio between the lining and the damping layer, may also influence the structural response and deserve further investigation. In addition, the present study assumes similar spatial variability characteristics (SOF) for the main random rock parameters, which represents a simplification adopted to maintain computational tractability under the probabilistic framework. In practice, different geotechnical parameters may exhibit different spatial correlation structures, and a more refined treatment of parameter-specific SOFs would further improve the robustness of the analysis. Although the material parameters of RSC were validated through comparison with available experimental results, the present study remains primarily a numerical investigation, and more comprehensive validation of the overall tunnel-scale dynamic model would benefit from additional field data or large-scale experimental evidence. Furthermore, more systematic quantitative comparisons between deterministic and probabilistic approaches, such as through fragility curves, reliability indices, and additional probabilistic response metrics, would further strengthen the engineering interpretation of the proposed framework.

From an engineering perspective, the probabilistic failure estimates presented in this study provide additional information beyond conventional deterministic analysis by quantifying the range of possible structural responses under seismic loading. This can help engineers better identify unfavorable design scenarios and assess the potential benefits of applying RSC damping layers in seismic regions. In particular, the proposed framework may provide a useful basis for comparing different damping-layer configurations, including thickness selection, under spatially variable geological conditions. Although the present results are not intended to directly define design code provisions or fixed safety factors at the current stage, they may provide a useful probabilistic basis for future performance-based tunnel design and more risk-informed engineering decisions.

## Figures and Tables

**Table 1:** Criteria of seismic failure grades.

Seismic Failure Grades	Relative Deformation Rate η (‰)
Null	<0.6
Minor	0.6–2.5
Severe	2.5–4
Extreme	>4

**Table 2:** Random field parameters of rock mass.

Parameter	$\mu_{X}$	COV*_X_*	SOF*_X_*		$\rho_{c,\varphi }$
			$\delta_{hX}$	$\delta_{vX}$	
*E*	4.00 GPa	43.12%	50.23 m	12.10 m	/
*ν*	0.3	12.03%			/
*c*	5.00 MPa	24.55%			−0.67
*φ*	39°	32.31%			

**Table 3:** Elastoplastic parameters of the C30 and RSC.

Material	Density	E	ν
C30	2400 kg/m^3^	30 GPa	0.2
RSC	1148 kg/m^3^	66.3 MPa	0.26

**Table 4:** Numerical sets for probabilistic analysis.

Set	PGA (g)	Tunnel Depths (m)
A1 (A1c)	0.2	300
A2 (A2c)	0.4	
A3 (A3c)	0.6	
A4 (A4c)	0.8	
A5 (A5c)	1.0	
A6 (A6c)	1.2	
B1 (B1c)	0.1	80
B2 (B2c)		100
B3 (B3c)		150
B4 (B4c)		200
B5 (B5c)		300

Note: The unbracketed cases represent the test groups with the RSC damping layer (thickness = 0.175 m), with bracketed cases indicating control groups using standard lining without seismic isolation layers.

## Data Availability

The original contributions presented in this study are included in the article. Further inquiries can be directed to the corresponding author.
